# Effectiveness and safety of ciprofol for the induction and maintenance of general anesthesia in urological surgery: a prospective, non-inferiority cohort study

**DOI:** 10.3389/fmed.2025.1590922

**Published:** 2025-09-16

**Authors:** Ling Zhan, Shuang Xie, Jing-xiao Lu, Fan Zhang

**Affiliations:** ^1^Department of Anesthesiology, East Hospital, Renmin Hospital of Wuhan University, Wuhan, Hubei, China; ^2^Department of Radiology, Xinhua Hospital, Shanghai Jiao Tong University School of Medicine, Shanghai, China

**Keywords:** ciprofol, propofol, induction, maintenance, urological surgery

## Abstract

**Introduction:**

Ciprofol is a newly developed intravenous agent, with limited clinical data available to date. The study aimed to evaluate the effectiveness and safety of ciprofol for general anesthesia in patients undergoing urological surgery.

**Methods:**

This study consecutively enrolled 172 urological patients aged ≥ 18 years who received general anesthesia. A total of 166 eligible patients were assigned to two groups: ciprofol (*n* = 85; induction 0.3–0.4 mg·kg^−1^; maintenance, 1.0–1.5 mg·kg^−1^·h^−1^) and propofol (*n* = 81; induction, 1.5–2.0 mg·kg^−1^; maintenance, 4–8 mg·kg^−1^·h^−1^). The primary effectiveness endpoint was the difference in anesthesia success rates between the two groups. The secondary effectiveness endpoints included the normal rate of the bispectral index (BIS), time to adequate sedation, time to loss of the eyelash reflex, diachronic changes in the BIS, mean arterial pressure (MAP), heart rate, recovery time, and extubation time. Adverse events (AEs) were recorded to evaluate the safety profiles of ciprofol.

**Results:**

The anesthesia success rate was 100% in both groups. The lower limit of the 95% confidence interval (CI) for the rate difference (RD) exceeded the prespecified non-inferiority margin of −10%. The time to adequate sedation and the time to loss of the eyelash reflex were longer with ciprofol compared to propofol (*p* < 0.001). The diachronic changes in the BIS and MAP in the ciprofol group decreased at a relatively slower rate during induction, indicating that ciprofol had a slower but smoother onset of action compared to propofol. The recovery time and extubation time were similar between the two groups. Ciprofol was associated with significantly lower incidences of injection pain, hypotension, and deep anesthesia compared to propofol. No patient in either group showed intraoperative awareness or postoperative cognitive decline.

**Conclusion:**

Ciprofol is non-inferior to propofol in terms of effectiveness and safety. It can be safely and effectively used for the induction and maintenance of general anesthesia in patients undergoing urological surgery.

## 1 Introduction

Since the 1980s, propofol has been the primary intravenous sedative used by anesthesiologists for the induction and maintenance of general anesthesia (1). Propofol provides adequate sedation (2) and has a very short terminal half-life, which allows for rapid recovery (3). However, propofol has a narrow therapeutic index and can cause significant cardiovascular and respiratory depression and hypoxia, which may occasionally necessitate emergency endotracheal intubation (4, 5). Propofol also commonly causes injection pain (6). Given an aging population, increasing demand for more comfortable general anesthesia, and limitations of existing agents, there is an urgent need to discover new anesthetics with higher potency and fewer adverse effects (7, 8). These demands, together with continuing advances in clinical pharmacology, have given rise to the concept of “soft” drugs—safer agents designed to have wider therapeutic indices and to undergo rapid, predictable metabolism into inactive metabolites (9).

Ciprofol is a new type of optically active 2,6-disubstituted alkylphenol compound—an (R)-configuration small-molecule isomer—that acts as a short-acting gamma-aminobutyric acid-A (GABA_A_) receptor agonist. Its mechanism of action involves the enhancement of GABA-mediated chloride influx, producing sedative and anesthetic effects (9). In some clinical trials, ciprofol has been shown to provide safe and effective sedation for the induction of general anesthesia, and its pharmacokinetic and pharmacodynamic profiles are similar to those of propofol (10, 11). Nevertheless, as ciprofol is newly developed, there are limited clinical data regarding its use for both the induction and maintenance of general anesthesia. Therefore, we conducted this trial to investigate the effectiveness and safety of ciprofol for the induction and maintenance of general anesthesia in patients undergoing urological surgery.

## 2 Materials and methods

### 2.1 Study design and procedures

This single-center, prospective, non-inferiority cohort clinical trial included participants from the Department of Urology between July and September 2022. Eligible participants were consecutively enrolled and assigned to two equally sized groups (ciprofol and propofol). Ethical approval (protocol number WDRY2022-K079) was obtained from the Ethical Committee of Renmin Hospital of Wuhan University, Wuhan, China, and the trial was registered with the Chinese Clinical Trial Registry Center (ChiCTR2300072767). All participants or their families provided informed consent before enrollment.

**Figure 1:**
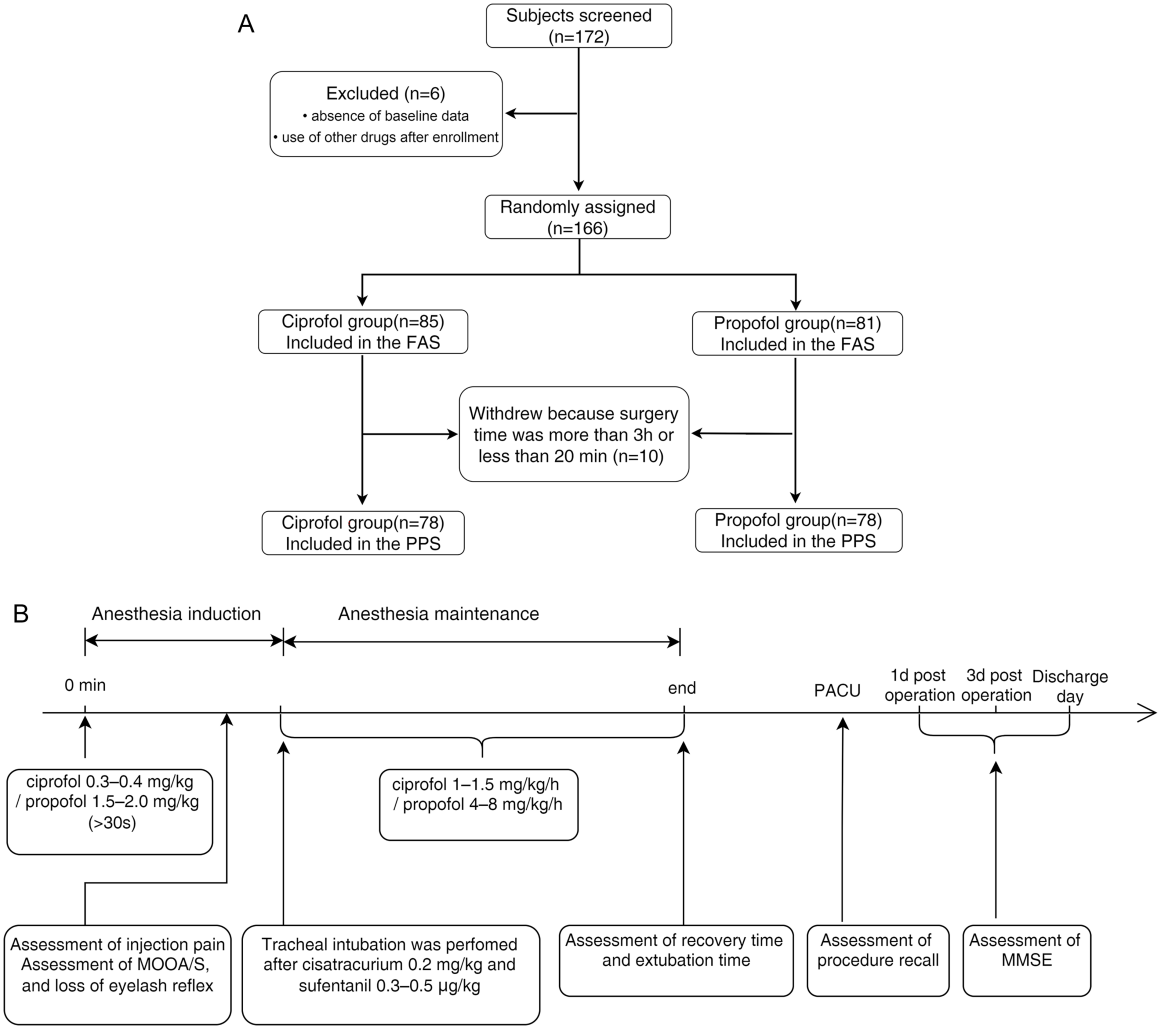
**(A)** Flow diagram. A total of 172 patients were screened for enrolment in this study. Of these, six patients were excluded because of missing baseline data or the post-enrolment use of other drugs. Therefore, 166 patients were included in the full analysis set (81 in the propofol group and 85 in the ciprofol group). A total of 10 participants were excluded from the study because the surgery duration was more than 3 h or was shorter than 20 min. Therefore, the per-protocol set analysis comprised 156 participants (78 in each group). **(B)** Schematic of this study. Ciprofol was administered at an induction dose of 0.3–0.4 mg·kg^−1^ and a maintenance dose of 1.0–1.5 mg·kg^−1^·h^−1^. Propofol was administered at an induction dose of 1.5–2.0 mg·kg^−1^ and a maintenance dose of 4–8 mg·kg^−1^·h^−1^.

The participants fasted for 6–8 h. Upon arrival in the operating room, baseline measurements were taken, including non-invasive blood pressure (NIBP), heart rate (HR), and pulse oxygen saturation (SpO_2_), and an electrocardiogram (ECG) was recorded. The Modified Observer’s Assessment of Alertness/Sedation (MOAA/S) scale (12) and the bispectral index (BIS) were used to monitor and evaluate the depth of anesthesia (13). Train-of-four stimulation modes were used to monitor the muscle relaxation effects. Baseline values were recorded 5 min before drug administration. Measurements were taken every 1 min for the first 20 min after anesthesia induction and, thereafter, at progressively longer intervals until the patients left the operating room. The flow diagram is shown in Figure 1A, and the study schema is presented in Figure 1B.

Based on previous research (14–16), either ciprofol (0.3–0.4 mg·kg^−1^; lot number: 20220106, Liaoning Haisco Pharmaceutical Co., Ltd., Liaoning, China) or propofol (1.5–2.0 mg·kg^−1^; lot number: SB324, AstraZeneca Ltd., Cambridge, United Kingdom) was administered as the induction dose over 30 s. Injection pain was assessed, and the time to adequate sedation was recorded, with MOAA/S scores recorded every 5 s from the start of induction until a score of ≤ 1 was reached. After the loss of consciousness, an endotracheal tube was inserted smoothly, followed by the administration of cisatracurium (0.2 mg·kg^−1^) and sufentanil (0.3–0.5 μg·kg^−1^).

After induction, the patients in each group received a continuous infusion of ciprofol (1.0–1.5 mg·kg^−1^·h^−1^) or propofol (4–8 mg·kg^−1^·h^−1^). Remifentanil was administered to both groups at 6–10 μg·kg^−1^·h^−1^. When the T1 value from neuromuscular monitoring returned to 25% of the baseline value, an additional dose of cisatracurium (0.02 mg·kg^−1^) was administered.

If signs of light anesthesia were identified —such as BIS > 65, an increase in mean arterial pressure (MAP) or HR by > 30% from baseline, lacrimation, or sweating—emergency treatment was promptly initiated. This involved administering a bolus of ciprofol (0.2 mg·kg^−1^) or propofol (0.5 mg·kg^−1^) for the respective group, and the infusion rate was increased to deepen the anesthesia. If these measures were ineffective, a bolus of rescue propofol (0.5 mg·kg^−1^) was administered based on the clinical judgment of the anesthesiologist. Anesthesia maintenance was considered a failure if rescue propofol was required.

All anesthetic agents were discontinued at the end of surgery, and no reversal agents were administered. Participants were extubated in the operating room and then transferred to the post-anesthesia care unit for observation for at least 30 min. During this time, the NIBP, ECG, SpO_2_, and respiratory rate were monitored, and intraoperative awareness/recall was assessed using the Modified Brice Questionnaire (17).

The dosage of sedatives, analgesics, and muscle relaxants, the type and dosage of intraoperative vasoactive drugs, and operation time were recorded. Adverse events (AEs) were also recorded to evaluate safety profiles. Cognitive changes were assessed using the mini–mental state examination (MMSE) scale (18) to evaluate postoperative cognitive decline.

### 2.2 Participant eligibility

The inclusion criteria were as follows: (1) male or female patients aged >18 years who were scheduled for elective surgery expected to last between 20 min and 3 h; (2) American Society of Anesthesiologists physical status *Ι*, II, or Ш; (3) body mass index of 18–30 kg·m^−2^; (4) voluntary provision of written informed consent; and (5) willingness to comply with study requirements and postoperative follow-up.

The exclusion criteria were as follows: (1) known allergy or hypersensitivity to the study drugs or their excipients; (2) long-term use of sedative or narcotic medications, alcohol consumption, or substance abuse; (3) pregnancy, planning to become pregnant within 1 month postoperatively, or breastfeeding at the time of participant screening; (4) clinically significant cardiovascular, respiratory, or renal disease; and (5) recent use of cytochrome P450 inhibitors or other clinical trial drugs during the screening period.

### 2.3 Clinical outcome assessments

The primary effectiveness endpoint was the success rate of anesthesia, defined as follows: (1) no intraoperative procedure recall, (2) no intraoperative movement, and (3) no need for rescue sedative drugs. The difference in the success rate of anesthesia between the groups—expressed as the rate difference (RD)—was calculated. A lower limit of the 95% confidence interval (CI) for the RD of less than −10% indicated that the curative effect of ciprofol was non-inferior to that of propofol. The non-inferiority margin (∆) was set at −10% based on the relevant regulatory guidelines (19–21).

The secondary effectiveness endpoints included the following: (1) differences in the rate of the normal BIS between the groups and the RD of the normal BIS, where a normal BIS was defined as 40 to 65; (2) time to adequate sedation, defined as the interval from the initial administration of the study drug to the first occurrence of an MOAA/S score of ≤ 1; (3) time to loss of the eyelash reflex, defined as the interval from the initial administration of the study drug to the first confirmed loss of the reflex; (4) diachronic changes in the BIS, MAP, and HR between the two groups, where diachronic changes emphasized the rate of change over time, especially from baseline to eight subsequent timepoints; (5) recovery time, defined as the interval from cessation of anesthetic administration to the first time an MOAA/S score > 4 was recorded on three consecutive occasions; and (6) extubation time, defined as the interval from cessation of anesthetic administration to extubation. All secondary effectiveness endpoints were analyzed in the participants whose anesthesia maintenance was successfully completed.

### 2.4 Safety assessments

Safety indicators were assessed by monitoring AEs and adverse drug reactions, mainly including the following: (1) bradycardia: HR < 50 beats·min^−1^ for ≥ 30 s; (2) tachycardia: HR > 100 beats·min^−1^ or an increase of ≥30% from baseline for more than 2 min; (3) hypertension: systolic blood pressure (SBP) > 180 mmHg, diastolic blood pressure (DBP) > 100 mmHg, or an increase of ≥30% from baseline for more than 2 min; (4) hypotension: MAP < 65 mmHg, SBP < 90 mmHg, DBP < 60 mmHg, or a decrease of ≥ 30% from baseline for more than 2 min to maintain stable vital signs to reduce complication (adverse event) rates; (5) injection pain: patient complaints of pain, an escape reflex, or obvious facial frowning during the bolus injection of the study drugs; (6) deep anesthesia: BIS < 40, MAP < 65 mmHg, and a simultaneous ≥ 30% decrease in MAP/HR from baseline during induction and maintenance; (7) intraoperative procedure recall: assessed using the Modified Brice Questionnaire within 10 min after the patient achieved full alertness (MOAA/S = 5), with any affirmative response (“Yes”) to the question “Do you remember anything between going to sleep and waking up?” considered procedure recall; and (8) postoperative cognitive decline: evaluated using the MMSE scale on postoperative days 1 and 3 and at discharge, with a diagnosis made when the postoperative score was ≥ 2 points lower than the preoperative baseline value.

Anesthesiologists were responsible for ensuring patient safety throughout the procedure and intervened only when necessary to maintain stable vital signs. The investigators’ role was limited to collecting experimental data, and they did not influence or alter the anesthesia management of any patient.

### 2.5 Sample size calculation and statistical analysis

The sample size was estimated based on the following assumptions: (1) the sedation success rate in the propofol control group; (2) a non-inferiority margin of −10%; and (3) a one-sided significance level of 0.025, with a power of 80%. Allowing for an estimated dropout rate of 10%, the total required sample size was 172 participants (86 in each group).

The experimental data were analyzed using SAS (version 9.4). The two-sample *t*-test was used for between-group comparisons of normally distributed data, and the Wilcoxon rank-sum test was applied for skewed data. The CMH-χ^2^ test or Fisher’s exact test was used for between-group comparisons of count data. The Newcombe–Wilson method was used to calculate the RD between the two groups. Differences were considered statistically significant at a *p*-value of < 0.05. Effectiveness analyses were performed on both the full analysis set (FAS) and the per-protocol set (PPS), while safety analyses were conducted using the PPS.

## 3 Results

### 3.1 Participant characteristics

A total of 172 participants were screened for enrolment in this study. Of these, six were excluded due to missing baseline data or the use of other drugs after enrolment. Consequently, 166 participants completed the safety assessment and were included in the FAS (81 in the propofol group and 85 in the ciprofol group). Precisely 10 participants withdrew because the surgery duration exceeded 3 h or was less than 20 min. Therefore, the PPS analysis included 156 participants (78 in each group) (Figure 1A).

Demographics and baseline characteristics (FAS) were compared between the two groups (Table 1). No statistically significant differences were observed between the two groups for any measured parameters.

**Table 1:** Baseline demographics and clinical hemodynamics (full analysis set).

Characteristic	Ciprofol (*n* = 85)	Propofol (*n* = 81)	*p-*value
Sex (*n*, %)			
Male	64 (75.3)	62 (76.5)	
Female	21 (24.7)	19 (23.5)	0.851
Age (years)	61.8 ± 9.2	61.1 ± 10.5	0.637
Median (Min, Max)	63 (30, 84)	62 (21, 82)	
BMI (kg·m^−2^)	23.7 ± 2.9	24.0 ± 3.1	0.553
ASA status (*n*, %)			
I	38 (44.7)	38 (46.9)	0.939
II	42 (49.4)	39 (48.2)	
III	5 (5.9)	4 (4.9)	
MAP (mmHg)	101.6 ± 11.1	99.1 ± 10.8	0.143
HR (beats·min^−1^)	78.1 ± 10.6	79.0 ± 10.1	0.553
BIS value	94 ± 4	94 ± 4	0.637
MMSE score			
Mean ± SD	29 ± 1	29 ± 1	0.413
Median (Min, Max)	30 (24, 30)	30 (25, 30)	

Data are expressed as mean ± standard deviation and median (min, max) or as numbers with percentages. Demographics, baseline hemodynamics, and baseline BIS/MMSE values were compared using Student’s t-test, the chi-squared test, or the Wilcoxon rank-sum test. No statistically significant differences were observed. BMI, body mass index; ASA, American Society of Anesthesiologists; MAP, mean arterial pressure; BIS, bispectral index; MMSE, mini–mental state examination.

### 3.2 Primary effectiveness outcome

The anesthesia success rates in both the ciprofol and propofol groups were 100%, yielding an RD of zero. All urologic surgeries were completed in all participants. No participant experienced procedure recall, required rescue sedatives, or exhibited physical movement. The lower limit of the 95% CI for the RD in the anesthesia success rate (−7.6 to 7.6% in the FAS; −8.3 to 8.3% in the PPS, Table 2) was above the non-inferiority margin of −10%.

**Table 2:** Comparison of anesthesia induction success rates between the groups.

Analysis set	Group (*n*)	Success rate (%)	Rate difference (%)	95% CI
FAS	Ciprofol (*n* = 85)	100	0	−7.6, 7.6
	Propofol (*n* = 81)	100		
PPS	Ciprofol (*n* = 78)	100	0	−8.3, 8.3
	Propofol (*n* = 78)	100		

The Newcombe–Wilson method was used to calculate the rate difference between the two groups. The non-inferiority margin was set at −10%. FAS, full analysis set; PPS, per-protocol set; CI, confidence interval.

### 3.3 Secondary effectiveness outcomes

The normal BIS rates, RDs, and 95% CIs for the two groups are shown in Table 3. In the PPS, the RD for the normal BIS was 11.5% (95% CI: −3.8–26.9%); the lower bound of the 95% CI (−3.8%) exceeded the prespecified non-inferiority margin of −10%. The trends observed in the FAS were consistent with those in the PPS.

**Table 3:** Normal rates, rate differences, and 95% CIs of the BIS between the groups.

Analysis set	Group (*n*)	Normal rate of BIS (%)	Rate differences (%)	95% CI (%)
FAS	Ciprofol (*n* = 85)	63.5	10.4	−4.5, 25.4
	Propofol (*n* = 81)	53.1		
PPS	Ciprofol (*n* = 78)	64.1	11.5	−3.8, 26.9
	Propofol (*n* = 78)	52.6		

The Newcombe-Wilson method was used to calculate the rate difference between the groups. The non-inferiority margin was set at −10%. A normal BIS was defined as a value between 40 and 65. CI, confidence interval.

**Figure 2:**
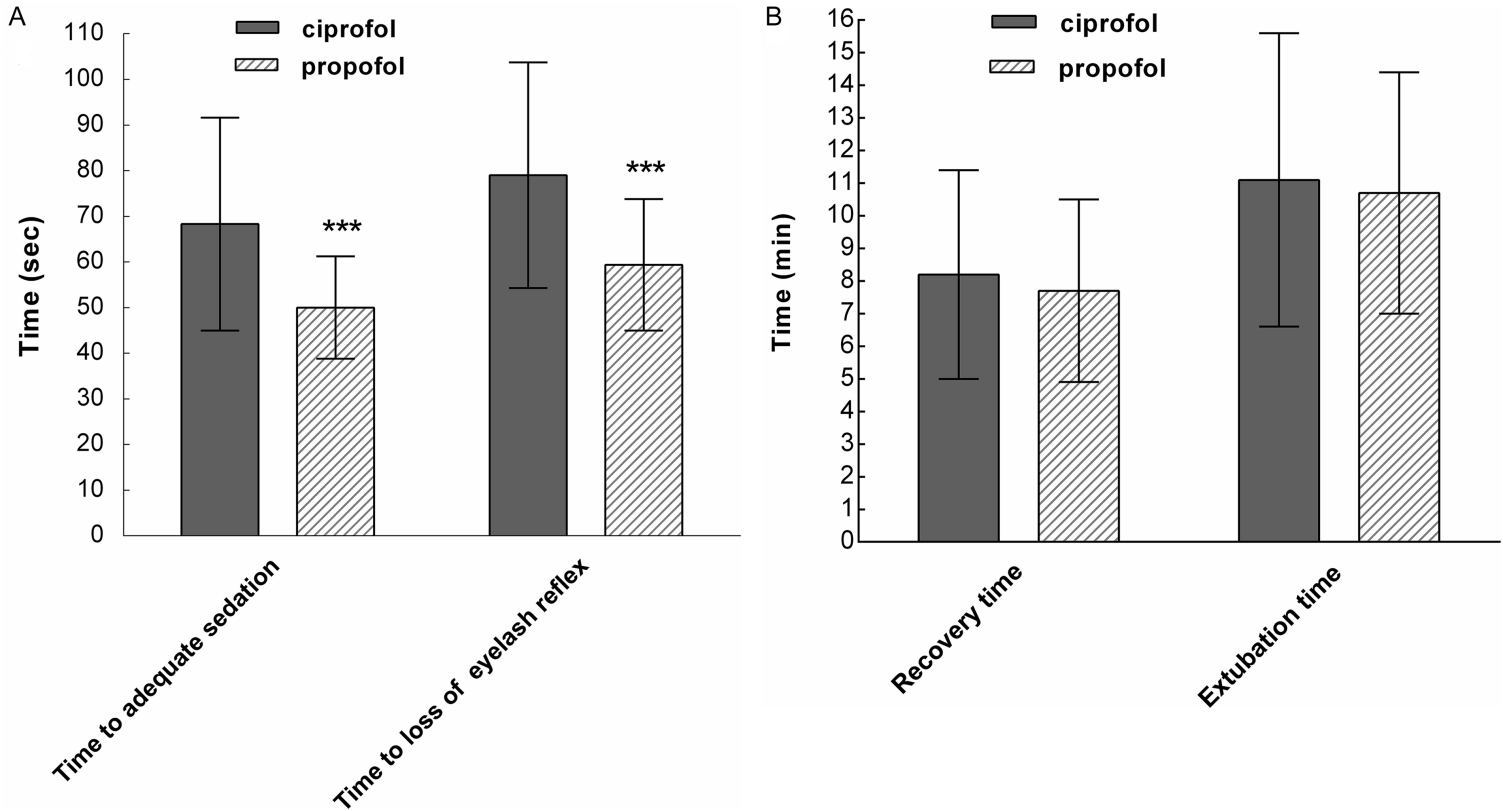
**(A)** The time to adequate sedation and the time to loss of the eyelash reflex were longer in the ciprofol group than in the propofol group (^***^p < 0.001). **(B)** Recovery time and extubation time were similar between the two groups (*p* > 0.05; per-protocol set).

The time to adequate sedation (MOAA/S score ≤ 1) was longer in the ciprofol group than in the propofol group (68.3 ± 23.3 s vs. 50.0 ± 11.2 s; *p* < 0.001). The time to loss of the eyelash reflex was also longer in the ciprofol group (79.0 ± 24.7 s vs. 59.4 ± 14.4 s; *p* < 0.001) (Figure 2A). Recovery and extubation times in the ciprofol group were slightly longer compared to the propofol group; however, these differences were not statistically significant (Figure 2B). The trends observed in the FAS were also consistent with those in the PPS.

**Figure 3:**
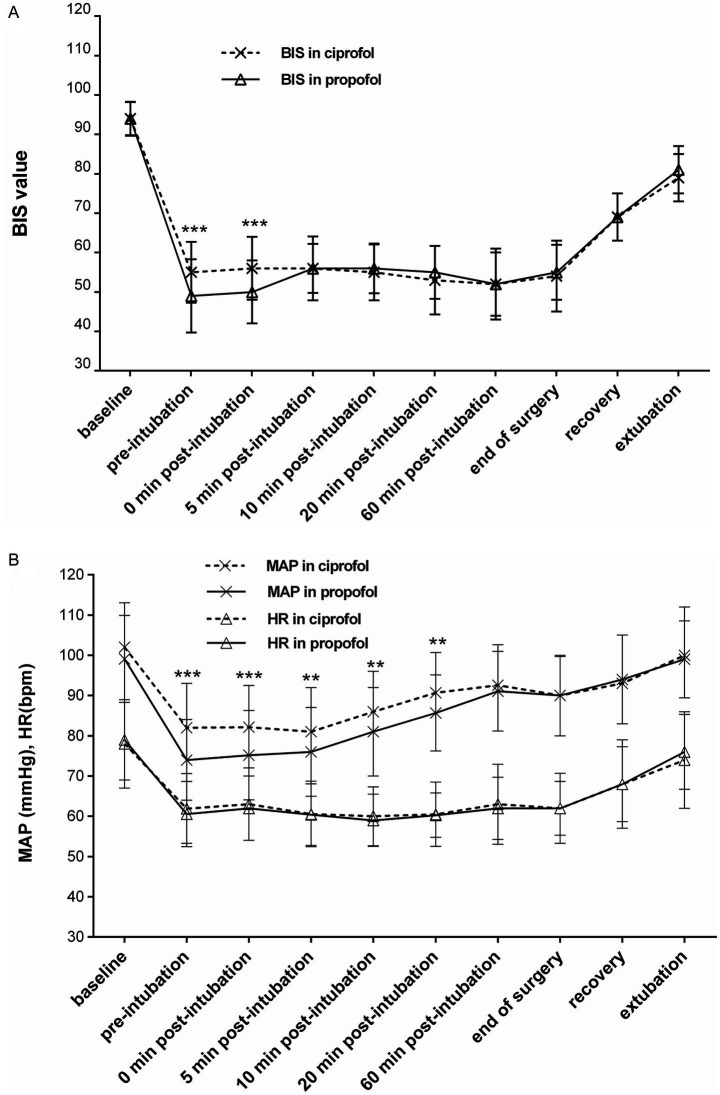
**(A)** Changes in the BIS during induction and maintenance [per-protocol set (PPS)]. Significant between-group differences were observed at pre-intubation and 0 min post-intubation (***p < 0.001). **(B)** Changes in mean arterial pressure (MAP) and heart rate (HR) during induction and maintenance (PPS). Differences in MAP between the groups were significant within 20 min after induction (***p < 0.001 to **p < 0.01), whereas no significant difference was observed in HR during the induction and maintenance phases.

The changes in the BIS, MAP, and HR during the induction and maintenance phases of the two groups are shown in Figures 3A,B, respectively. Differences in the BIS were significant during the induction phase (*p* < 0.001), and differences in MAP were significant at certain time points within 20 min after induction (*p* < 0.001 to *p* < 0.01). There was no significant difference in HR between the two groups during either the induction or maintenance phase. The diachronic changes in the BIS and MAP are shown in Table 4. Through the diachronic changes, we found that the BIS and MAP in the ciprofol group decreased at a slower rate from baseline to pre-intubation and from baseline to 0 min post-intubation (Table 4). In contrast, the BIS increased significantly in the propofol group from 0 min to 5 min post-intubation compared to the ciprofol group (propofol: 49.8 → 56.4; ciprofol: 56.3 → 55.8) (Figure 3A). The diachronic changes in HR were similar between the two groups. The trends observed in the FAS were consistent with those in the PPS.

**Table 4:** Diachronic changes in the BIS and MAP between the two groups (per-protocol set).

	BIS				MAP			
Time period	Ciprofol (*n* = 78)	Propofol (*n* = 78)	Wilcoxon statistic	*p*-value	Ciprofol (*n* = 78)	Propofol (*n* = 78)	Wilcoxon statistic	*p-*value
Baseline to pre-intubation	39.41 ± 7.76^***^	44.96 ± 10.25^***^	3.49	<0.001^†††^	20.72 ± 11.14^***^	25.50 ± 8.92^***^	3.020	0.002^††^
Baseline to 0 min post-intubation	37.44 ± 8.34^***^	43.71 ± 9.56^***^	1.273	0.092 ^†^	20.00 ± 11.18^***^	23.79 ± 9.02^***^	2.421	0.015 ^†^
Baseline to 5 min post-intubation	38.26 ± 8.78^***^	37.30 ± 7.14^***^	−0.620	0.535	21.19 ± 11.85^***^	22.57 ± 9.74^***^	1.042	0.297
Baseline to 10 min post-intubation	39.51 ± 7.72^***^	37.97 ± 7.31^***^	−1.175	0.240	16.23 ± 11.71^***^	17.96 ± 10.58^***^	0.897	0.370
Baseline to 20 min post-intubation	40.46 ± 8.45^***^	39.32 ± 8.16^***^	−0.518	0.604	12.08 ± 12.27^***^	12.88 ± 9.30^***^	0.678	0.498
Baseline to 60 min post-intubation	41.07 ± 9.26^***^	42.23 ± 9.57^***^	0.632	0.528	11.98 ± 12.80^***^	7.82 ± 9.37^***^	2.051	0.040 ^†^
Baseline to end of surgery	25.03 ± 7.54^***^	24.77 ± 6.75^***^	−0.046	0.963	9.30 ± 13.69^***^	4.60 ± 9.63^***^	1.843	0.065 ^†^
Baseline to recovery	15.63 ± 6.86^***^	12.45 ± 7.34^***^	−2.880	0.004^††^	2.14 ± 13.23	0.05 ± 9.09	0.864	0.387

Diachronic changes emphasize the change rate during a given time period and are expressed as mean ± standard deviation. The BIS and MAP in the ciprofol group decreased at a slower rate during induction. The Wilcoxon rank-sum test was used for comparisons between the groups. The Wilcoxon signed-rank test was used for comparisons within a group. ^†^*p* < 0.05, ^††^*p* < 0.01, ^†††^*p* < 0.001. * indicates comparisons within a group for the changes from baseline to each time point, ^*^p < 0.05, ^**^p < 0.01, ^***^p < 0.001. BIS, bispectral index; MAP, mean arterial pressure.

There were no significant between-group differences in operation time and in the doses of sufentanil, cisatracurium, and remifentanil. However, the consumption of vasoactive agents (dopamine) was significantly higher in the propofol group than in the ciprofol group in the PPS (16.5 ± 7.9 mg vs. 8.2 ± 4.6 mg; *p* < 0.01).

### 3.4 Safety assessment

A total of eight participants in the ciprofol group and six in the propofol group experienced bradycardia under general anesthesia (*p* > 0.05). The incidence of hypotension was reported in 18 patients (23.1%) in the ciprofol group and 42 patients (53.8%) in the propofol group (*p* < 0.001). Injection pain occurred in one participant (1.3%) in the ciprofol group and 45 participants in the propofol group (57.7%, *p* < 0.001). Furthermore, 14 participants (17.9%) in the propofol group experienced “deep anesthesia,” while none in the ciprofol group did (*p* < 0.001). None of the participants responded “Yes” to the Modified Brice question “Do you remember anything between going to sleep and waking up?” indicating that no patient in either group had intraoperative awareness (Table 5). The MMSE scores on postoperative days 1 and 3 and at discharge did not differ significantly between the groups. Overall, no patient withdrew from the study because of adverse reactions, and no serious adverse reactions occurred.

**Table 5:** Summary of adverse events (per-protocol set).

Adverse events	Ciprofol (*n* = 78)	Propofol (*n* = 78)	Statistic	*p*-value
Bradycardia (*n*, %)	8 (10.3%)	6 (7.7%)	0.314	0.575
Hypotension (*n*, %)	18 (23.1%)	42 (53.8%)	15.600	<0.001
Injection pain (*n*, %)	1 (1.3%)	45 (57.7%)	59.687	<0.001
Deep anesthesia (*n*, %)	0	14 (17.9%)	15.380	<0.001
Intraoperative awareness (*n*, %)	0	0		
Myoclonus (*n*, %)	2 (2.5%)	0	Fisher	0.497
Vomiting (*n*, %)	1 (1.3%)	1 (1.3%)	Fisher	1.000
Tachycardia (*n*, %)	0	0		

Data are expressed as numbers with percentages. Hypotension, injection pain, and “deep anesthesia” within 20 min after induction occurred significantly less frequently with ciprofol.

## 4 Discussion

As a novel, short-acting GABA_A_ receptor agonist, ciprofol is a novel choice for achieving smooth and comfortable anesthesia. In the present study, we analyzed the effectiveness and safety of ciprofol for general anesthesia induction and maintenance in patients undergoing elective urological surgery.

During the induction and maintenance, all participants in the ciprofol group achieved a 100% anesthesia success rate and did not require top-up doses or rescue drugs. The lower limit of the 95% CI for the RD in the anesthesia success rate and normal BIS rate exceeded the prespecified non-inferiority margin of −10%, indicating that ciprofol, at induction and maintenance doses of 0.3–0.4 mg·kg^−1^ and 1.0–1.5 mg·kg^−1^·h^−1^, respectively, can provide sufficient anesthesia and is non-inferior to propofol at an induction dose of 1.5–2.0 mg·kg^−1^ and a maintenance dose of 4–8 mg·kg^−1^·h^−1^.

In the present study, the sedation level was assessed using the MOAA/S scale, which captures not only the responsiveness component of the original scale (awake [5]–unresponsive [0]) but also the response to painful stimuli. Although anesthesia blocks reactions to verbal commands, reactions to painful stimuli may persist. An MOAA/S score of ≤ 1 was used to indicate adequate sedation or successful induction. In our study, the time to adequate sedation (MOAA/S ≤ 1) and the time to loss of the eyelash reflex were longer in the ciprofol group than in the propofol group. Although we did not measure serum drug concentrations, our findings suggested that ciprofol at 0.3–0.4 mg·kg^−1^ had a slightly slower onset of anesthetic action than propofol at 1.5–2.0 mg·kg^−1^ when used for anesthesia induction. These results are consistent with those of some previous studies (11, 14, 16, 22). However, other studies (23–25) reported no between-group differences in the time to successful induction or the time to loss of the eyelash reflex. In these studies, the induction doses of ciprofol and propofol were 0.4 and 2.0 mg·kg^−1^, respectively. Nevertheless, during the pilot study, we found significant circulatory depression with these induction doses, especially among older patients. To maintain stable vitals, we adjusted the induction dose of ciprofol to 0.3 mg·kg^−1^ and propofol to 1.5 mg·kg^−1^ for participants aged >65 years, and kept the doses of ciprofol at 0.4 mg·kg^−1^ and propofol at 2.0 mg·kg^−1^ constant for participants aged ≤65 years. Although age was not the sole factor considered, it served as a practical and immediate criterion, as older patients are generally more prone to comorbidities and hemodynamic instability. The threshold of 65 years was selected based on clinical convention in China, where it represents the typical retirement age and an inflection point in overall health status. Following this adjustment, the incidence of hypotension during induction significantly reduced. The dose adjustment resulted in differences in the timing metrics. However, despite the time to adequate sedation and the time to loss of the eyelash reflex being slightly prolonged in the ciprofol group, the average time to successful anesthesia induction was still approximately 1 min in both groups. These results indicated that ciprofol induces effective sedation with a rapid onset of action, slightly slower than propofol, without affecting the clinical anesthesia process.

We found that the time to loss of the eyelash reflex was generally longer than the time to adequate sedation in the ciprofol group, with an average lag of approximately 15 s. By contrast, in the propofol group, loss of the eyelash reflex generally coincided with the achievement of adequate sedation (MOAA/S score was ≤ 1). This result differs from those of previous studies (16, 23, 26), which demonstrated that the time to loss of the eyelash reflex was shorter than the time to attainment of an MOAA/S score of ≤ 1. A possible reason is that those studies administered premedication—0.04 mg·kg^−1^ midazolam and 0.3 μg·kg^−1^ sufentanil—before anesthesia induction, which may interfere with the assessment of the study drugs’ onset. To avoid such interference in the present study, we did not give any additional sedatives prior to the initial administration of the study drugs until adequate sedation was achieved (MOAA/S score was ≤ 1).

Diachronic change emphasizes the rate of change over a period rather than absolute numerical values at specific time points. Through diachronic changes, we observed that the BIS and MAP decreased more slowly during induction in the ciprofol group. Conversely, the BIS increased significantly in the propofol group within 5 min post-intubation compared to the ciprofol group. Although the difference in recovery time between the two groups was not statistically significant, it was approximately 1 min longer in the ciprofol group than in the propofol group. Within 20 min after induction, the incidence of AEs, such as hypotension and “deep anesthesia,” differed significantly by group: hypotension occurred in 18/78 (23.1%) of the ciprofol participants versus 42/78 (53.8%) of the propofol participants, and deep anesthesia occurred in 0/78 (0.0%) of the cipofol participants versus 14/78 (17.9%) of the propofol participants. Consistent with these findings, dopamine consumption in the propofol group was significantly higher than that in the ciprofol group.

Injection pain is a commonly reported adverse reaction following propofol administration (4, 6, 27). Consistent with previous reports, the rate of injection pain in our study was significantly lower in the ciprofol group than in the propofol group (1.3% vs. 57.7%). This difference may be related to the concentration of free drug in the aqueous phase of the injection solution. Ciprofol’s greater lipid solubility and lower dosing result in a lower concentration of free drug in the aqueous phase of the injection solution compared to propofol injection; this pharmacological profile may contribute to the lower incidence of injection pain (28–30). The relative rarity of injection pain with ciprofol may be particularly advantageous in pediatric anesthesia, where reduced procedural pain can lessen perioperative stress and improve patient comfort.

Postoperative MMSE scores did not differ between the two groups at any assessed point. Cognitive function assessed by the five MMSE domains (orientation, registration, attention and calculation, recall, and language) showed minimal changes across both groups. By discharge, all participants had returned to normal cognitive and memory function, as assessed by the MMSE scale.

In the present study, diachronic change analysis indicated a slower rate of change and a more stable anesthetic profile for ciprofol during induction. The combined use of the MOAA/S score and BIS monitoring improved the assessment of anesthesia depth.

Although our findings strongly suggest that ciprofol exhibits a slightly slower yet smoother onset of anesthetic effects, it also appears to provide a more stable sedation level than propofol, potentially because of its slower metabolic rate. With these unique advantages, ciprofol may be more suitable for older and critically ill patients, as well as those with multiple comorbidities, thereby offering benefits in clinical practice.

This study has some limitations. It was a single-center clinical investigation, and the study duration was limited to less than 3 h. The study population was not stratified by age (younger adults: 18–65 years; older adults: ≥65 years). In addition, no blood samples were collected, preventing the monitoring of plasma concentrations of ciprofol and propofol. Future research should include a more detailed comparison of ciprofol and propofol in older adults and in individuals with comorbidities. Such studies should evaluate plasma concentrations, dose-effect correlations, metabolic rates, degree of drug accumulation during longer surgical procedures, and the potential influence of age, ideally through multicenter, double-blinded clinical trials.

## 5 Conclusion

Ciprofol is non-inferior to propofol in terms of effectiveness and safety, as it was associated with milder circulatory depression and reduced injection pain in this study. It can be safely and effectively used as an alternative for both the induction and maintenance of general anesthesia in patients undergoing urological procedures.

## Data Availability

The raw data supporting the conclusions of this article will be made available by the authors, without undue reservation.
